# Delivering clinical tutorials to medical students using the Microsoft HoloLens 2: A mixed-methods evaluation

**DOI:** 10.1186/s12909-024-05475-2

**Published:** 2024-05-04

**Authors:** Murray Connolly, Gabriella Iohom, Niall O’Brien, James Volz, Aogán O’Muircheartaigh, Paschalitsa Serchan, Agatha Biculescu, Kedar Govind Gadre, Corina Soare, Laura Griseto, George Shorten

**Affiliations:** 1 Cork University Hospital and University College Cork, Cork, Ireland; 2 University College Cork, Cork, Ireland; 3 Cork University Hospital, Cork, Ireland

**Keywords:** Mixed Reality, HoloLens, Medical Education, Augmented Reality

## Abstract

**Background:**

Mixed reality offers potential educational advantages in the delivery of clinical teaching. Holographic artefacts can be rendered within a shared learning environment using devices such as the Microsoft HoloLens 2. In addition to facilitating remote access to clinical events, mixed reality may provide a means of sharing mental models, including the vertical and horizontal integration of curricular elements at the bedside. This study aimed to evaluate the feasibility of delivering clinical tutorials using the Microsoft HoloLens 2 and the learning efficacy achieved.

**Methods:**

Following receipt of institutional ethical approval, tutorials on preoperative anaesthetic history taking and upper airway examination were facilitated by a tutor who wore the HoloLens device. The tutor interacted face to face with a patient and two-way audio-visual interaction was facilitated using the HoloLens 2 and Microsoft Teams with groups of students who were located in a separate tutorial room. Holographic functions were employed by the tutor. The tutor completed the System Usability Scale, the tutor, technical facilitator, patients, and students provided quantitative and qualitative feedback, and three students participated in semi-structured feedback interviews. Students completed pre- and post-tutorial, and end-of-year examinations on the tutorial topics.

**Results:**

Twelve patients and 78 students participated across 12 separate tutorials. Five students did not complete the examinations and were excluded from efficacy calculations. Student feedback contained 90 positive comments, including the technology’s ability to broadcast the tutor’s point-of-vision, and 62 negative comments, where students noted issues with the audio-visual quality, and concerns that the tutorial was not as beneficial as traditional in-person clinical tutorials. The technology and tutorial structure were viewed favourably by the tutor, facilitator and patients. Significant improvement was observed between students’ pre- and post-tutorial MCQ scores (mean 59.2% Vs 84.7%, *p* < 0.001).

**Conclusions:**

This study demonstrates the feasibility of using the HoloLens 2 to facilitate remote bedside tutorials which incorporate holographic learning artefacts. Students’ examination performance supports substantial learning of the tutorial topics. The tutorial structure was agreeable to students, patients and tutor. Our results support the feasibility of offering effective clinical teaching and learning opportunities using the HoloLens 2. However, the technical limitations and costs of the device are significant, and further research is required to assess the effectiveness of this tutorial format against in-person tutorials before wider roll out of this technology can be recommended as a result of this study

**Supplementary Information:**

The online version contains supplementary material available at 10.1186/s12909-024-05475-2.

## Introduction

Clinical tutorials which include encounters with real patients are recognised as integral elements in medical education [1–3]. Sir William Osler famously stated that “medicine is learned by the bedside and not in the classroom.” [4] However, many medical schools are facing challenges in delivering clinical education to students in an environment where there are increasing numbers of students, a limited number of patients and tutors, and increased scrutiny regarding the costs and environmental impacts of travel [5–8]. The COVID-19 pandemic also had a significant impact on in-person medical education in many countries, where students’ access to patients was severely curtailed [9, 10]..

The argument that medical education requires interactive tutorials on actual patients is supported by various educational theories. Bandura’s Social Learning Theory and Social Cognitive Theory propose that students learn via attention, retention, reproduction and motivation [11, 12]. This supports the need for direct observation and modelling of relevant clinical role-models participating in doctor-patient interactions [13, 14]..

The Constructivist theory is based on the premise that the act of learning is based on a process which connects new knowledge to pre-existing knowledge [15, 16]. Vertical Integration in medical education involves the integration of aspects of the curriculum across time, namely the integration of basic sciences and clinical sciences [17–19]..

Providing medical education within these frameworks, prioritising student exposure to direct interactions with clinicians and patients, and vertical integration of curriculum material, in situations where physical access to patients may be limited by numbers, logistics or infection control concerns poses a significant challenge to medical schools around the world. Utilising technology to facilitate the delivery of clinical education remotely may present a solution to these issues.

The broadcast of bedside tutorials to a remote location can be delivered using a “third-person” perspective, via a fixed or mobile broadcasting device, or using a first-person perspective, via a device mounted on the tutor. Devices which provide a first-person perspective are typically head-mounted-display devices (HMDs). The capabilities of these devices range widely, from basic two-way communication with a remote location, to devices with Augmented Reality (AR) and Mixed Reality (MR) functions which allow the integration of holographic artefacts into tutorials.

Augmented reality (AR) is a virtual environment that allows the user to view both their physical environment and virtual elements in real-time. Mixed Reality (MR) is an extension of AR which allows the real and holographic elements to interact [20, 21]..

The use of AR and MR are expanding in many industries including healthcare, education, engineering, and manufacturing [22–24]. MR investigated in a variety of settings pertaining to medical education. Many early studies focused on teaching relevant anatomy, and more recently studies have evaluated the use of MR in procedural training, and its use in streaming of clinical ward-rounds to medical students [25–33]..

Head-mounted-display devices which offer MR experiences are growing in number and capability [34].The Microsoft HoloLens2 is one such device which enables the creation of an immersive Mixed Reality environment and can superimpose holographic images onto the user’s surroundings.

The HoloLens 2 has a number of specific capabilities which can be utilised in the virtual delivery of in-person clinical tutorials.The device can facilitate educationally effective, three-way communication between students, tutors and patients, as well as facilitating the incorporation of mixed reality elements into tutorials. The MR capabilities may provide a means of sharing holographic artefacts such as images and diagrams, which can allow the vertical and horizontal integration of curricular elements at the bedside.Utilisation of the MR capabilities of the device may improve student experiences and learning, in particular through instructional scaffolding (e.g rendering cell, organ or system pathways proximate to a patient) [35] Given the device’s connectivity capabilities, students can be in a separate geographical location to the patient and tutor. This has the potential to decrease student travel requirements and enables the delivery of tutorials to students in multiple different locations simultaneously [36]. The tutorial can also be delivered to a greater number of students than would be practical in a traditional bedside clinical tutorial environment. This can decrease the burden on both tutors and patients in comparison to multiple smaller group sessions. Finally, infection control risks are reduced as only the tutor enters patients’ environments.

### Study goals

There is little published research to date which robustly evaluates the use of the HoloLens in replicating bedside tutorials while also incorporating mixed reality elements into the tutorials. The aims of this study are to evaluate the use of the Microsoft HoloLens 2 device to deliver a tutorial on preoperative anaesthetic history and upper airway examination to medical students in a remote location, while incorporating MR holograms in the tutorial delivery. Specific objectives include evaluating the feasibility of delivering tutorials with the HoloLens device, assessing the learning efficacy of these tutorials, and assessing student, tutor, facilitator, and patient perspectives of the tutorials.

## Methods

This study was approved by the Clinical Research Ethic Committee of the Cork Teaching Hospitals, and the University College Cork Research and Postgraduate Affairs Committee. All participants including students, patients, tutor and technical facilitator provided written informed consent prior to inclusion in the study.

### Study population

University College Cork medical students from two cohorts, third year Graduate-Entry and fourth year Direct-Entry medical students attending a tertiary referral teaching hospital for a clinical attachment with the Department of Anaesthesia and Intensive Care Medicine were invited to participate in the study. Both groups are in their second-last year of medical training, and thus have completed modules and examinations in basic medical sciences and clinical practice in the preceding years, with a maximum of 1 week experience in the field of anaesthesia [37–39]. Patients attending Cork University Hospital for scheduled surgery were selected and approached for consent by tutors according to clinical relevance. All participants were 18 years or over and were deemed capable of providing consent. Each student provided information on their age, gender and previous third-level qualifications.

### Tutorial Sturcture

**Fig. 1:**
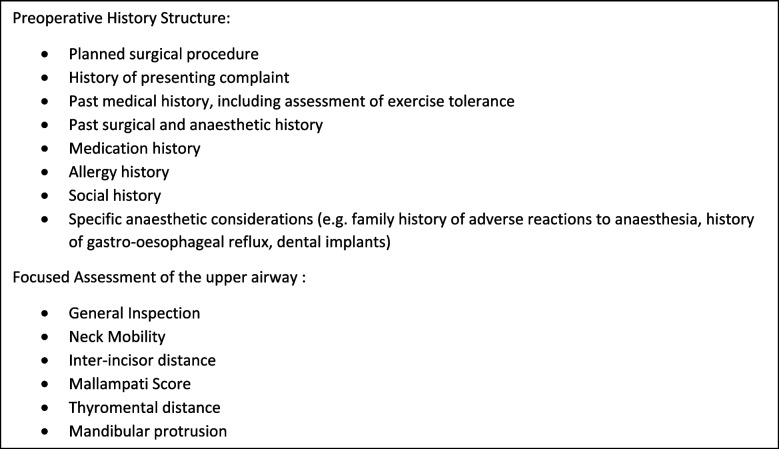
Preoperative Anaesthetic History and Focused Preoperative Assessment of the upper airway tutorial structure

A one-hour tutorial focusing on completing a preoperative history and focused assessment of the upper airway was developed by MC (adjunct clinical lecturer), GI (Senior Clinical Lecturer) and GS (Professor) in line with the University curriculum’s learning objectives. (Fig. 1) Tutorials were delivered on a weekly basis to groups of third year Graduate Entry and fourth year Direct Entry medical students across the 2021–2022 academic year.

All tutorials were delivered by one tutor (MC) and assisted by a technical facilitator (NOB), both males aged in their thirties, who enabled the connection between the site of the clinical encounter and nearby tutorial room. The tutor had no prior experience with the HoloLens 2 device or other AR HMDs prior to participation in this study; the facilitator had significant experience in its use. The tutor was given a period of familiarisation with the device which included using the Microsoft “HoloLens Tips” app, which provides a structured tutorial on the various hand gestures used to control the device, as well as a number of practice calls in order to test the network and audiovisual equipment in the tutorial room [40]. This familiarisation period totalled approximately 3 hours.

During the tutorial, the tutor (MC) interacted with a patient (face to face) in the pre-or postoperative units and remotely with a small group of [6–10] students in a nearby tutorial room. The remote interaction occurred via Hololens 2 worn by the tutor, institutional Wi-Fi (Eduroam), and Microsoft Teams.He demonstrated and explained the techniques of preoperative history taking and preoperative upper airway assessment.

Throughout the patient assessment the tutor interacted both with the patient and with the students as if conducting an in-person tutorial, providing additional information, asking the students pertinent questions, and expanding on the findings of the patient’s history and physical examination. Students communicated with the patient by asking questions via the tutor.

### Resources employed

Resources necessary to provide the tutorials via the HoloLens included capital costs of the HoloLens device (€3500) and microphone (€88) as well as annual licence costs of €275 per user (*n* = 4). Human resources employed in developing the tutorials and trialling equipment included approximately 20 hours of training, remote assistance (Microsoft) and collaboration between the tutor (MC), Professor (GS) and facilitator (NOB), as well as 5 hours input from the Senior Clinical Lecturer (GI).

### Internet connectivity

An internet connection of at least 1.5mpbs of bandwidth is recommended by Microsoft for best audio, visual and content sharing experience [41]. Secure, password protected wireless internet access via the University institutional network (Eduroam) was utilised by both tutor and students.

### Hardware

In most tutorials, broadcasts were hosted by an MSI running the Windows 10 operating system, audio was amplified using a Bose SoundLink Mini portable speaker and video was screened via a HDMI cable to a 36″ monitor. In one tutorial students accessed the tutorial via their personal smartphones or laptops. In order to bypass the noise cancellation technology within the HoloLens an external microphone (Saramonic SmartMic+UC L/weight Smartphone Mic USB-C) and 3.5 mm earphone were used.

### Software

**Fig. 2:**
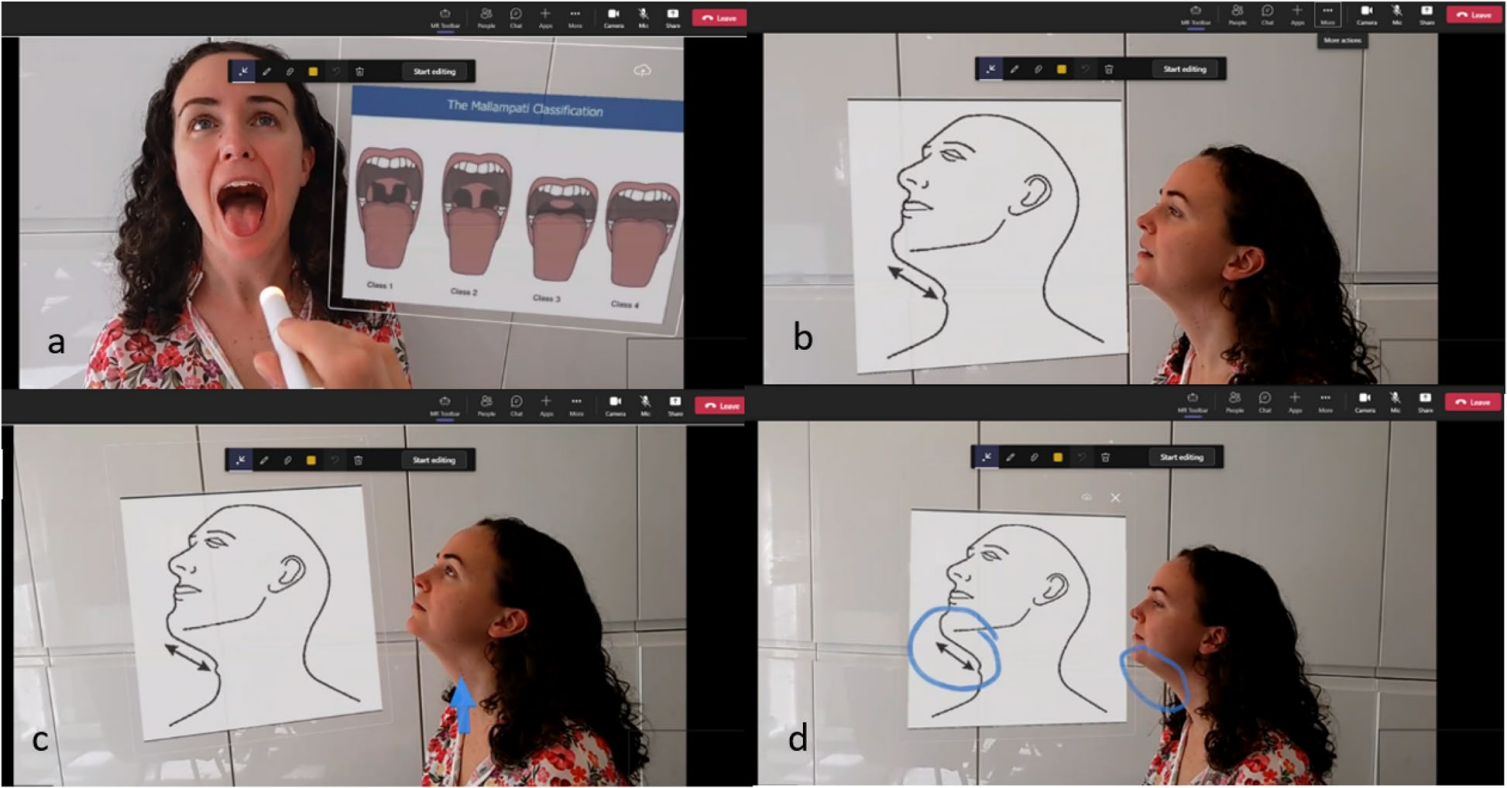
**a** Assessment of Mallampati Score. **b** Assessment of Thyromental Distance. **c** Identification of thyroid cartilage using holographic pointer. **d** Illustration of holographic “drawing” function

Dynamics 365 Remote Assist application was used, in-tandem with Microsoft Teams, to host each video call. This connection allowed the students to see the tutors field of vision and hear both the tutor and patient. Hand gestures including the “hand-ray”, “air-tap”, “air-tap and hold” and “start-gesture” were used to control the HMD and manipulate the holographic artefacts. Relevant holographic artefacts were superimposed during the tutorial. This included the insertion of diagramatic representaions of the Mallampati scoring system and Thyromental Distance during the airway assessment portion of the tutorial [Fig. 2 (a) and (b)]. The holographic pointer and “drawing” functions were used by the tutor to highlight relevant upper airway structures and emphasise information on the holographic diagrams [Fig. 2 (c) and (d)].

### Assessment of tutor perceptions

Immediately after completion of the first tutorial, the tutor completed a System Usability Scale assessment and on completion of the last tutorial, the tutor and facilitator summarised their perceptions of using the HMD.

### Assessment of student perceptions

Immediately after completion of the tutorial, students completed a modified Evaluation of Technology-Enhanced Learning Materials: Learner Perceptions (ETELM-LP) questionnaire in order to assess their perceptions of the tutorial, which incorporated a seven-point Likert Scale and open questions [42]. Cronbach’s Alpha was calculated after exclusion of question 1 and reverse scoring of questions 13 and 15.

Three students also took part in semi-stuctured interviews via Microsoft Teams. Researchers undertook this study from an interpretive approach [43]. The interviews were conducted by JV, and followed a template of questions and corresponding probes from which the interviewer expanded as appropriate [Additional file 1]. The template served as a foundation from which the interviewer expanded as appropriate. The interviews were recorded and transcribed. Analysis of the interview transcripts and questionnare responses was performed using Dedoose Qualitative Research Software Version 4.3.Qualitative data from interviews and feedback questionnaires were coded thematically in alignment with Clarke and Braun’s suggestions for qualitative analysis [44]. Following the initial thematic coding, researchers conducted a content analysis to strengthen the interpretation of results. Illustrative quotes were chosen based on the representativeness of the theme or subtheme and the clarity of their intrinsic interpretation. In alignment with current literature, the quotes selected were determined to be illustrative of the point, reflective of patterns observed, and relatively succinct [45]..

### Assessment of patient perceptions

On completion of the tutorials, patients were also asked to complete a mixed quantitative and qualitative questionnaire in order to assess their perceptions of the tutorial.

### Assessment of learning efficacy

We carried out a prospective non-comparative study of tutorial efficacy. Students completed a pre-tutorial Multiple Choice Question (MCQ) examination to assess baseline knowledge [Additional file 2], and a post-tutorial MCQ two to 3 days later [Additional file 3]. Students then completed an end-of-year assessment two to 5 months later consisting of a data interpretation exam and an Objective Structured Clinical Examination (OSCE) which focused on preoperative history taking and preoperative assessment of the upper airway respectively [Additional files 4 and 5]. These examinations were written by an investigator and the University Senior Clinical Lecturer in line with University standards. Examination results were converted to percentages and the data interpretation and OSCE results were combined to give a total End-of-Year result.

The Chi-Squared test was used to compare direct-entry and graduate-entry student demographics. Welch’s two-sample t-Test assuming unequal variances was used to compare student group ages. The Shapiro Wilk and Kolmogorov-Smirnov Tests were used to assess to normality of distribution of student assessment scores for data sets less than 50 and greater than 50 respectively. The Mann-Whitney U Test was performed to compare group performance in assessments and overall student performance between the pre- and post-tutorial examinations, and between the post-tutorial and End-of-Year scores. Cohen’s d was calculated for the pre and post-tutorial MCQ scores to assess effect size.

## Results

Twelve tutorials were completed involving 12 separate patients and 78 students. Four students did not complete the post-tutorial MCQ and one did not complete the End-of-Year assessments due to illness related absences. These students were excluded from efficacy calculations. Baseline characteristics of the student participants are summarised in Table 1. As expected the graduate-entry students was a significantly older cohort (graduate-entry median age 26 vs direct-entry mean of 22). Mean age of patient participants was 43.25, with an SD of 16.48, and a range of 18–64.

**Table 1:** Baseline characteristics of student participants

	Direct Entry Medical Students	Graduate Entry Medical Students	Total Students	*p*-Value
Number of Students n (%)	46 (58.9)	32 (41.1)	78	
Male n, (%)	14 (30.4)	14 (43.75)	28 (35.9)	*P* = 0.27
Age median (IQR [range])	22 (21–22 [20–33])	26 (25–28 [22–35])	23 (22–26[20–35])	*P* < 0.05

### Feasibility

We found that it was feasible to use the HoloLens2 to facilitate weekly bedside tutorials on live patients in a busy, tertiary referral teaching hospital. No tutorials were cancelled or postponed due to technology-related issues. Of note, in order to improve the audio quality of the patient’s voice, it was neccessary to add the USB microphone, which is not routinely supplied with the HoloLens 2. The tutorials were also dependent on secure Wi-Fi access for both tutor and students, the presence of a tutorial facilitator to control the equipment at the student end, and access to a quiet space to examine the patient.

### Tutor feedback

The sole tutor (MC) completed the System Usability Scale score, which was 72.5 (a score > 68 is deemed above average). The tutor (MC) stated that the HoloLens 2 was found to be comfortable to wear, the visor was unobtrusive and did not interfere with interaction with the patient or impede visualisation of clinical signs. The interaction with the device via hand gestures was relatively smooth and intuitive after the intial familiarisation period and the MR functions including the insertion of holographic diagrams, pointing, drawing and highlighting were useful. The holographic artefacts were visible throughout the tutorials at a “brightness” setting of seven out of 10.

Occasionally when talking to the students via the HMD, it was not clear to the patient if the tutor was talking to the patient or to the students. Utilising a structured pattern of speech such as “I am now talking to the students” was found to be useful to overcome this issue.

### Facilitator feedback

The technical facilitator (NOB) found that the set-up of the live broadcast to the students was akin to that of a video presentation and that the learning curve for hosting the tutorials was short as the Dynamic 365 Remote Assist application was quite similar to general videoconferencing software. He noted that patient proximity to the tutor was essential to ensure adequate audio quality and referenced an example where a supine patient was farther from the device than normal and that patient responses had to be repeated by the tutor. Backgound noise was noted as a “minor issue and transient in nature”, and the technical facilitator accepted that a certain amount of background noise was unavoidable in an active hospital ward.

### Student feedback

**Fig. 3:**
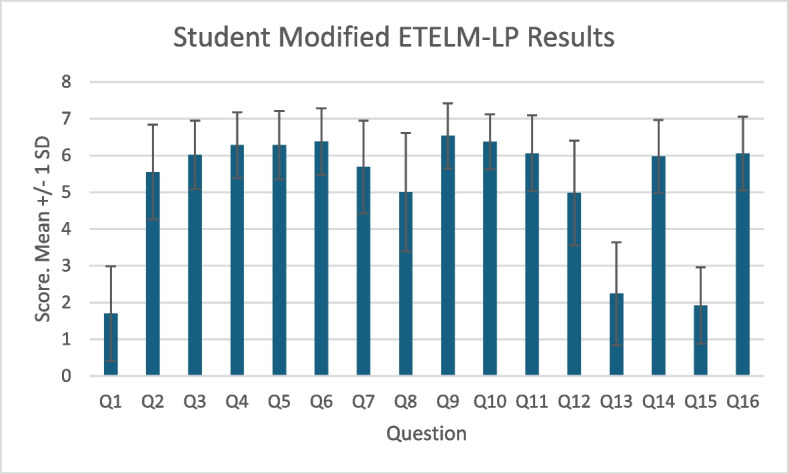
Student Modified ETELM-LP Scores. 7 point Likert scale with 7 as strongly agree and 1 as strongly disagree. Presented as Mean +/− 1 Standard Deviation

Quantitative student feedback via the modified ETELM-LP questionnaire is summarised in Fig. 3. Results are presented as (mean, SD) and refer to a seven-point Likert scale. Students had little experience in MR prior to the tutorial (1.7, 1.29). They found the audio and visual quality was clear and that the MR elements of the tutorial were useful. Most agreed the tutorial approximated a live patient encounter (5.69, 1.26), was more beneficial than a PowerPoint-based tutorial, and were neutral when asked if it was as beneficial as a live clinical encounter (5, 1.69). They did not agree that the tutorial structure required inappropriately high technology skill levels on the part of the students, nor that the MR elements served as a distraction. Most agreed that they would like MR to be incorporated into further tutorials (6.05, 1). Cronbach’s Alpha, excluding question 1 was calculated as 0.86, displaying good internal consistency.

### Student qualitative feedback results

Analysis of written and verbal feedback from 78 students identified 90 specific positive excerpts and 62 negatives (Table 2). Positive feedback included the technology’s ability to broadcast the tutor’s point-of-vision, the inclusion of holographic artefacts, and the remote nature of the tutorial. Negative feedback included issues with the audio-visual stream quality, the fact that students were not able to individually carry out the practical examination, and 11 students expressed concerns that the tutorial was not as useful as traditional in-person bedside clinical tutorials.

**Table 2:** Student feedback qualitative results

	Number of statements identified, n	Illustrative quotes
**Positive Themes**		
Praise for the technology	29	“Having the examiner’s perspective was important, so being able to see what they saw, and you know, being able to also see what they were looking for from their perspective.”
The device’s role as an adjunct for teaching	28	“Very helpful, especially for larger groups of students when not feasible to go to bedside.” “very beneficial to use … if a situation like COVID-19 happened, where we couldn’t attend placement.”
Favourable outlook on the session design	12	“That’s one place that the HoloLens does better than the bedside teaching. You bring [the diagrams] up and see it, rather than on bedside teaching recall it off memory or have to go back on your phone.”
**Negative Themes**		
Comments on the design of the session	29	“It would be good to have a go at some of the practical things like measuring a thyromental distance but otherwise was a good tutorial.”
Technical problems encountered	25	“The video playback was choppy.” “I had some issues with hearing the patient responses.”
Potential barriers to student learning	12	“Not as useful as bedside teaching.” “Does not substitute hands on time.”

Three students participated in semi-structured interviews. The limited sense of “presence” and interaction with the patient were identified as limitations to the format by all three interviewees. With respect to the physical examination one student explained he would have preferred to “experience it yourself, and have a look and feel and touch”. Specific mention was made of the value of combining broadcast (patient) and rendered (schematics) images, “The adding of the images … right next to the patient was really, really helpful”. This may indicate the potential to employ this format to support vertical and horizontal integration of curricular elements. All three interviewed students reported either a six or seven (on a verbal scale of 1–7) when asked to recommend this technology for inclusion in the medical curriculum.

### Patient feedback

**Fig. 4:**
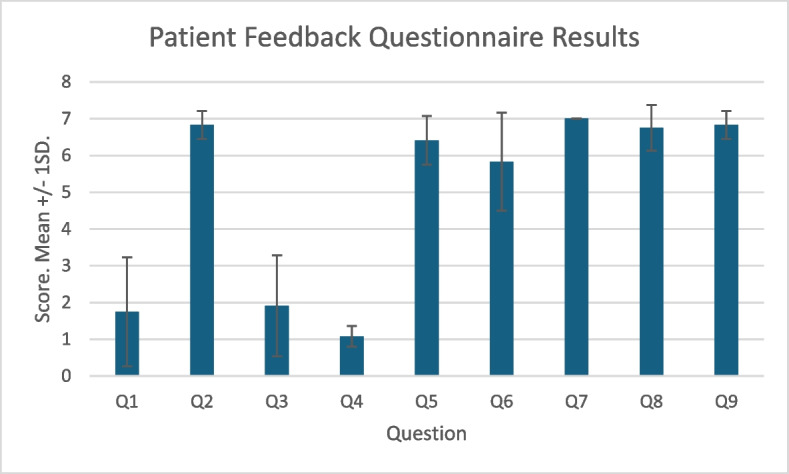
Patient Feedback Questionnaire Results. 7 point Likert scale with 7 as strongly agree and 1 as strongly disagree. Presented as Mean +/− 1 Standard Deviation

Quantitative feedback data from patient questionnaires is summarised in Fig. 4. Most patients had little experience with MR in the past (mean, SD: 1.75, 1.48) apart from one patient who scored 6. All agreed that the communication with the tutor was clear, that they felt safe, that the experience was enjoyable and that they would participate in a similar session in the future. Six of seven expressed that it was preferable to both small (5 or less) and large group in-person tutorials. Most patients did not agree that the HoloLens served as a distraction or made them uncomfortable.

Five patients gave qualitative feedback. Positive comments included that “it is good to see that you are moving on with new technology”, “it was well explained beforehand so I was very comfortable” and “it was fantastic to teach students when they can’t be at the bedside. Very unobtrusive”. One patient commented that “sometimes not sure if he [the tutor] was talking to me or the students” and another commented that “it would be lovely to see who I was talking to [the student group]”.

### Learning efficacy

**Fig. 5:**
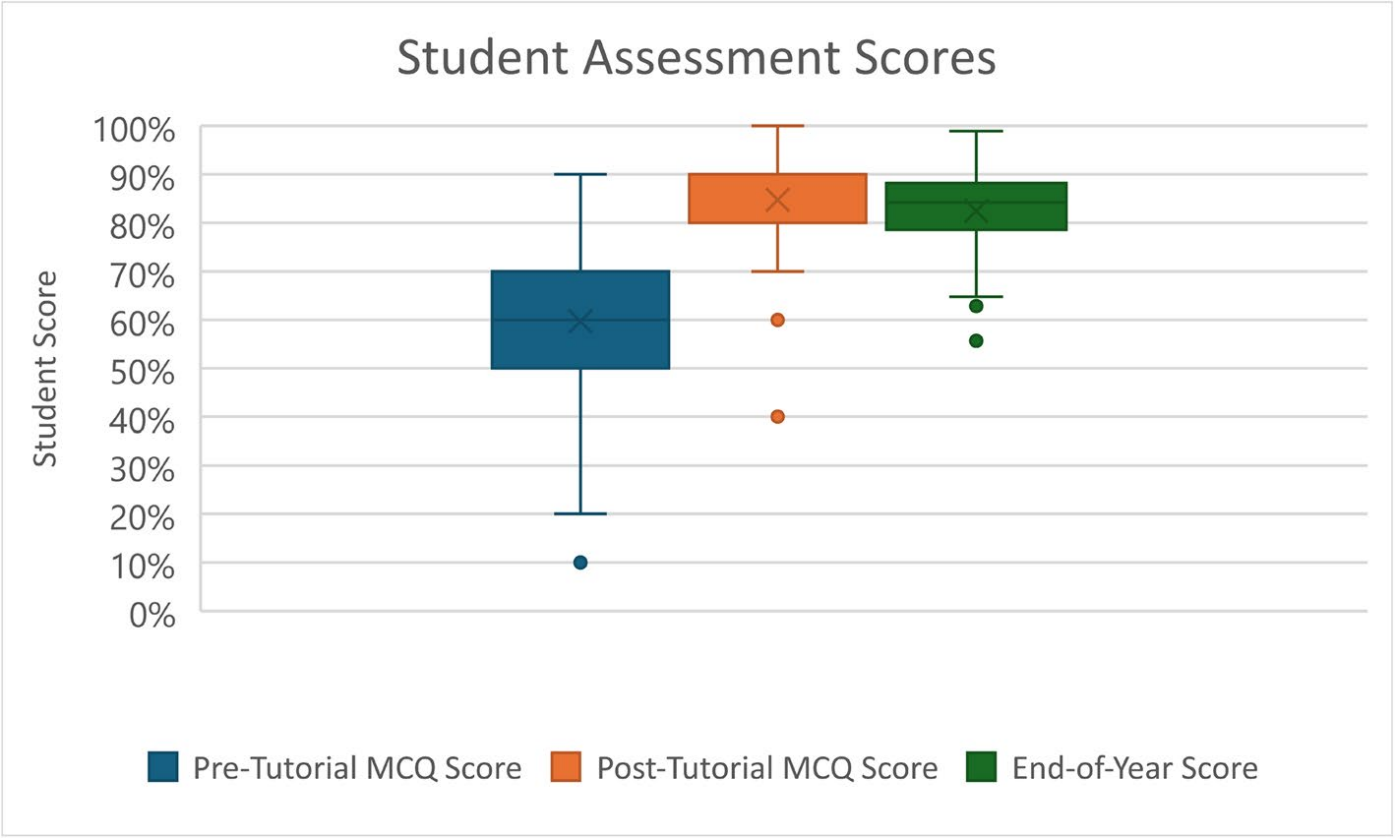
Boxplot of overall student assessment scores

Student examination scores are sumarised in Table 3 and Fig. 5. Student assessment scores were not normally distrubuted. A statistically significant improvement was observed between overall students’ pre and post tutorial MCQ scores (mean 59.2% Vs 84.7%, *p* < 0.001). Cohen’s d was 0.612, indicating a medium effect size. There was a statistically significant difference in student performance between the post tutorial MCQ and the composite End-of-Year scores (84.7% Vs 82.2%, *p* < 0.05). There were no statistically significant differences found between the graduate-entry and direct-entry students for any individual examination.

**Table 3:** Student Assessment Scores. Values are mean percentage score (Standard Deviation)

	Total % (SD)	Direct-Entry Students % (SD)	Graduate-Entry Students % (SD)	*p*-value
Pre-Tutorial MCQ	59.2 (18.5)	62.4 (17)	57.7 (18.6)	*p* = 0.21
Post-Tutorial MCQ	84.7 (11.7)	85.9 (12.9)	83.5 (9.8)	*p* = 0.16
Data Interpretation	76.3 (14.8)	78.1 (12.3)	74.8 (16.8)	*p* = 0.66
OSCE	88.3 (10.4)	89.2 (11.1)	87 (9.7)	*p* = 0.7
Total End-of-Year	82.2 (8.5)	83.6 (7.6)	80.9 (9.0)	*p* = 0.18

## Discussion

Mixed Reality headsets offer several novel capabilities which can facilitate remote education and vertical and horizontal integration of curriculum elements, particularly when aligned with appropriate educational theories such as Constructivism and Social Cognitive Theory. A large number of studies have focused on applying the technology in surgical and anatomical subject fields [46]. However, there are significant gaps in the evidence base, particularly studies specific to anaesthesiology, clinical exam, and addressing the provision of interactive tutorials to remote locations. Our study has demonstrated that it is feasible and effective to use the Microsoft HoloLens 2, incorporating its Mixed Reality functions to provide a live bedside tutorial on anaesthetic preoperative assessment to students situated in a remote location. Feedback from students, patients and the tutor were generally positive. Quantitative feedback from students regarding the audio-visual quality was mainly positive, however technical issues were noted, and preference for in-person tutorials was expressed by a minority of students.

Mill et al. previously examined the feasibility of the HoloLens 2 in broadcasting medical ward rounds [26]. While papers such as that by Mill et al. demonstrated the feasibility of utilizing the HoloLens 2 HMD to stream educational ward-rounds, they did not utilize the MR functions of the HMD, nor assess the learning efficacy of the device [26]. This study incorporates both quantitative and qualitative feedback from multiple sources, namely students, patients, the tutor, and tutorial facilitator. We believe this demonstrates a robust examination of the perceptions of the relevant stakeholders involved in the provision of clinical tutorials to medical students. Our findings that the tutorials were feasible, agreeable to both patients and students, and that students had occasional audio-visual difficulties are consistent with those of Mill et al. Our study additionally demonstrates that incorporation of holographic artefacts is both feasible and regarded by the tutor and students as useful, and that the tutorials provide effective knowledge acquisition.

Our tutorial format aimed to reproduce some of the educationally relevant components of an in-person tutorial. Other suggested structures advocate streaming video of the physician as opposed to the physician’s point-of-view [47]. The HoloLens 2 device allows the students to view the tutor’s field of vision which we argue is superior, and student feedback reflected this. This viewpoint allows students to appreciate in real time the clinical signs demonstrated during the clinical examination and correlate these with the holographic diagrammatic examples used. The MR environment provides an ideal setting to facilitate vertical integration in real time by displaying holographic artefacts of anatomical, physiological and pathological information, as well as patient specific data such as radiological imaging or lab results while interacting with a patient. Furthermore, delivering tutorials remotely reduces infection-control concerns and allows delivery to greater numbers of students in multiple locations.

Preserving patient confidentiality is essential in medical practice and education. In our study, both the HMD and devices at the student end were connected to secure institutional Wi-Fi and accessed via University accounts. Also, access to the audio-visual stream was controlled by the technical facilitator, and the students were located in a supervised tutorial room. It would be essential to control both access to the tutorial and the environment to which it is broadcast to maintain confidentiality.

### Limitations

Our study design has a number of limitations. It is non-comparative, and thus we are unable to draw conclusions regarding the relative learning experience or efficacy associated with tutorials delivered via the HoloLens device and the more traditional in-person bedside tutorials. Additionally, the different assessment methods between the MCQs and end of year examinations make direct measurement of knowledge retention difficult. The number of patients involved in the study was relatively small, and thus interpretation of both quantitative and qualitative data must be viewed in this context, and the generalisability of the data is low. The feedback from the tutor and tutorial facilitator must be viewed in the context that they were study investigators.

There are a number of limitations specific to research involving the HoloLens. Common limitations in studying the learning effects of the HoloLens in tested roles include the absence of validated measures and comprehensive evaluation instruments. Unlike other technologies, there are no benchmarks, datasets, or standard standardized protocols to specifically evaluate augmented reality systems, experiences, and methodologies [48–50]. Although the viewpoint offered to the students by the HoloLens allows the students to appreciate what the tutor is demonstrating, one drawback to this is that the focus of attention is primarily controlled by the tutor, and thus it is difficult for the tutorial to challenge the students to select the relevant areas to attend to. Depending on the tutorial topic and structure, an ideal virtual format may provide three perspectives: the tutors view, a third person view of the clinical encounter, and where applicable, an instrument’s view.

Regarding the generalisability of our study to other tutorial topics, the appreciation of clinical signs which would require palpation or auscultation would be beyond the current capabilities of the HoloLens 2 and therefore, careful tutorial design and topic selection is necessary.

## Conclusions

Our results demonstrate the feasibility of facilitating remote bedside tutorials on preoperative anaesthetic assessment using the HoloLens 2. The tutorial structure was found to be agreeable to students, patients, and tutors. Provision of tutorials in the format described in this study may be an option for situations where students’ access to live bedside tutorials are limited. However, further research is required to characterise the role, potential and limitations of incorporating Mixed Reality into clinical medical education in a broader context. Poor audio-visual quality and lack of hands-on practice were found to be the most frequent issues identified in our study and may be significant limitations to the use of this technology in wider medical education. There are significant costs involved in developing the infrastructure and expertise necessary to provide tutorials in this format. Prior to this technology being adopted by educational institutions, we recommend the completion studies to compare the learning efficacy of MR facilitated remote tutorials and traditional in-person bedside tutorials.

### Supplementary Information

**Additional file 1.**

**Additional file 2.**

**Additional file 3.**

**Additional file 4.**

**Additional file 5.**

## Data Availability

The datasets used and/or analysed during the current study are available from the corresponding author on reasonable request.

## Abbreviations

AR: Augmented Reality
ETELM-LP: Evaluation of Technology-Enhanced Learning Materials: Learner Perceptions
HMD: Head-Mounted Display
IQR: Interquartile Range
MCQ: Multiple Choice Question
MR: Mixed Reality
OSCE: Objective Structured Clinical Examination
SD: Standard Deviation
