# Supplementary material for: Delivering clinical tutorials to medical students using the Microsoft HoloLens 2: A mixed-methods evaluation
Source: BMC Med Educ. 2024 May 4;24:498. doi: 10.1186/s12909-024-05475-2 (PMC11070104; doi:10.1186/s12909-024-05475-2)
Supplement: Supplementary file 1 — Additional file 1. [file 12909_2024_5475_MOESM1_ESM.docx]

Additional File 1

Qualitative Interview Structure

Cluster 1: Demographics

1. **Have you used mixed reality technologies like this before? Yes/no.**
   1. *[Probe] If yes, can you tell me a little bit more about this? When was this, and how was it used?*

1. **Before this session, how comfortable were you performing a preoperative anaesthetic history and airway exam?**
   1. *Could you express that on a scale of 1-7? (1= not comfortable, 7= very)*

Cluster 2:

1. **How was your experience of the HoloLens tutorial?**
   1. *[Probe:] What are your thoughts on the design and layout of the elements on the screen?*
2. **Can you tell me what you enjoyed the most about this experience?**
3. **Can you tell me what you found challenging during the tutorial?**
4. **What surprised you about the use of the HoloLens and Mixed Reality to deliver bedside teaching in this manner?**

Cluster 3: General Perception

1. **Think back to previous bedside tutorials you have had. What differences did you notice between your previous experiences and this one?**
   1. *[Probe:] What aspects of the HoloLens instruction were the most different from in-person teaching?*
2. **Was the HoloLens used effectively? Did it affect your learning? Please elaborate.**
3. **What did you think of the images used in the tutorial during the airway assessment?**
4. ***How do you feel the session affected your knowledge of the preoperative exam?***
   - 1. *[Probe:] Do you feel that this session was a valuable way to spend your time?*
     2. *[Probe:] How would you compare the benefit of this session to an in-person training session?*
5. **After this session, how comfortable were you performing a preoperative anaesthetic history and airway exam?**
   - 1. *Could you express that on a scale of 1-7? (1= not comfortable, 7= very)*
6. **On a scale from 1 to 7, how likely are you to recommend the HoloLens for inclusion in medical education? (1=not at all likely, 7=very likely)**
