# Supplementary material for: Delivering clinical tutorials to medical students using the Microsoft HoloLens 2: A mixed-methods evaluation
Source: BMC Med Educ. 2024 May 4;24:498. doi: 10.1186/s12909-024-05475-2 (PMC11070104; doi:10.1186/s12909-024-05475-2)
Supplement: Supplementary file 2 — Additional file 2. [file 12909_2024_5475_MOESM2_ESM.docx]

Additional File 2

Pre-tutorial Multiple Choice Questionnaire

1.
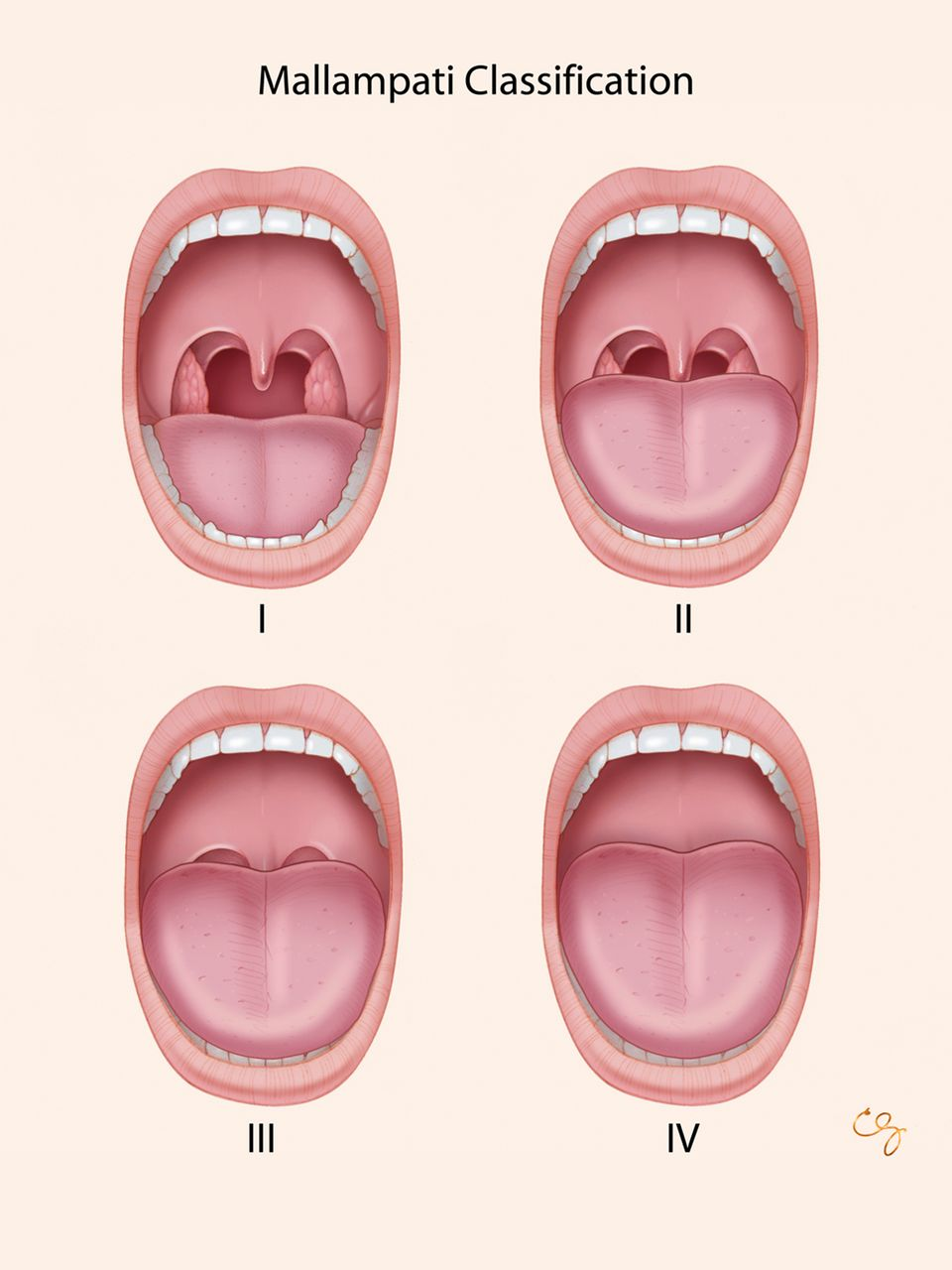


With regard to airway assessment, which term best describes this image?

1. Mallampati 1
2. Mallampati 2
3. Mallampati 3
4. Mallampati 4
5. Mallampati classification is not applicable

2.
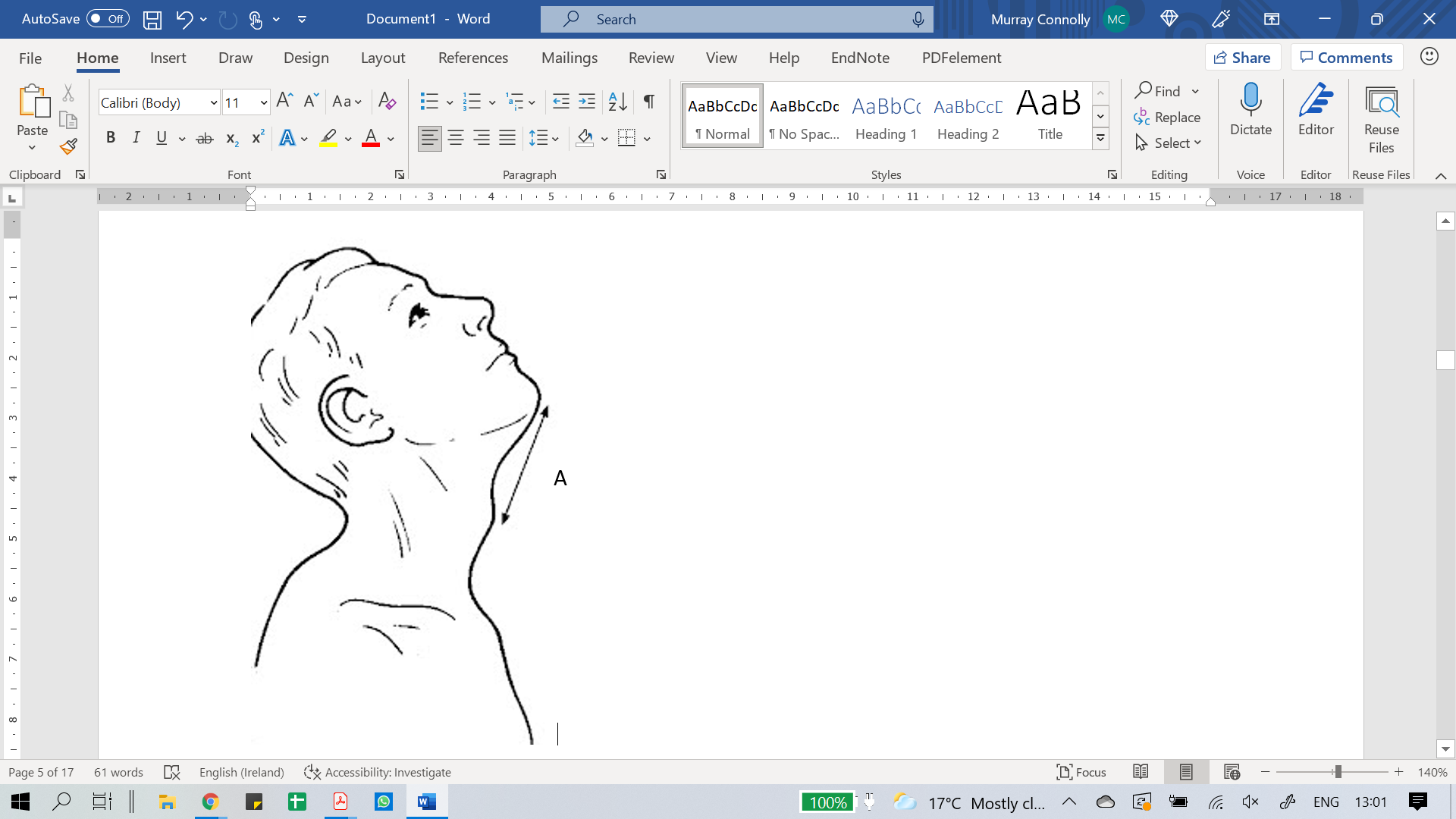


Regarding airway assessment, which sentence best describes the classical cut-off which may predict difficult airway management?

1. Distance A less than 6 centimetres
2. Distance A less than 8 centimetres
3. Distance A greater than 4 centimetres
4. Distance A greater than 6 centimetres
5. Distance A greater than 8 centimetres

3. Regarding airway assessment, which sentence best describes the classical cut-off which may predict difficult airway management?

1. Inter-incisor distance less than 4 centimetres
2. Inter-incisor distance less than 6 centimetres
3. Inter-incisor distance less than 8 centimetres
4. Inter-incisor distance less than 10 centimetres
5. Inter-incisor distance greater than 6 centimetres

4. Your next patient on the trauma list is a 23 year old man who fractured his forearm in a hurling match. He has well controlled asthma for which he takes a salbutamol inhaler occasionally. He has previously had an uneventful GA for a laparoscopic appendicectomy when he was 7. What is his ASA Classification?

- 1. ASA1
  2. ASA2
  3. ASA 2E
  4. ASA 3
  5. ASA 3E

5. You are assessing a 60 year old lady in the Pre-Anaesthetic Assessment Clinic. Which one of the following comorbidities is associated with difficulties in airway management?

1. Hypertension
2. Ischaemic Heart Disease
3. Ankylosing Spondylitis
4. Hypercholesterolaemia
5. Anaemia

6. You are the surgical intern in the Pre-Anaesthetic Assessment Clinic. You are assessing a 75 year old man scheduled for a lumbar decompression. Which of the following comorbidities is **not** commonly associated with difficulties in airway management?

1. Rheumatoid Arthritis
2. Macroglossia
3. BMI >40
4. Obstructive Sleep Apnoea
5. Ischaemic Heart Disease

7. A 64-year-old man is scheduled for a laparoscopic low-anterior resection. He has a past medical history of gastro-oesophageal reflux disease, hypertension and ankylosing spondylitis. On airway examination, what finding would be the most likely?

1. A Malampatti score of 4
2. A low thyromental distance
3. Reduced neck mobility
4. Reduced inter-incisor distance
5. Inability to prognath

8. You are the surgical intern in the Pre-Admission Assessment Clinic. You are assessing a seventy-year-old woman who is scheduled for an elective shoulder arthroscopy. She has a history of ischaemic heart disease, hypertension, gastrooesophageal reflux disease and diabetes mellitus. On questioning she states that she feels short of breath when mobilising short distances around her house. What would an estimate of her maximum METs be?

1. <4
2. 6
3. 4-10
4. >10
5. METs do not apply in this case

9. You are the surgical intern admitting a patient for an elective lumbar discectomy. He notes that he has a number of allergies. Which of the following is NOT one of the top-five most common causes of intra-operative anaphylaxis?

1. Patent Blue dye
2. Chlorhexidine
3. Depolarising Muscle Relaxants
4. Ondansetron
5. Penicillin

10. You are the surgical intern admitting a patient for an elective lumbar discectomy. She reports that the last time she underwent a general anaesthetic for a laparoscopic appendicectomy she was told afterwards that she had “aspirated after being put asleep” which necessitated a prolonged post-operative hospital stay. Which of the following conditions would NOT commonly increase the risk of aspiration of gastric content after induction of anaesthesia?

1. Abdominal pathology
2. Gastro-oesophageal reflux disease
3. Poorly controlled COPD
4. Emergency surgery
5. Pregnancy
