# Supplementary material for: Delivering clinical tutorials to medical students using the Microsoft HoloLens 2: A mixed-methods evaluation
Source: BMC Med Educ. 2024 May 4;24:498. doi: 10.1186/s12909-024-05475-2 (PMC11070104; doi:10.1186/s12909-024-05475-2)
Supplement: Supplementary file 3 — Additional file 3. [file 12909_2024_5475_MOESM3_ESM.docx]

Additional File 3

Post-tutorial Multiple Choice Questionnaire


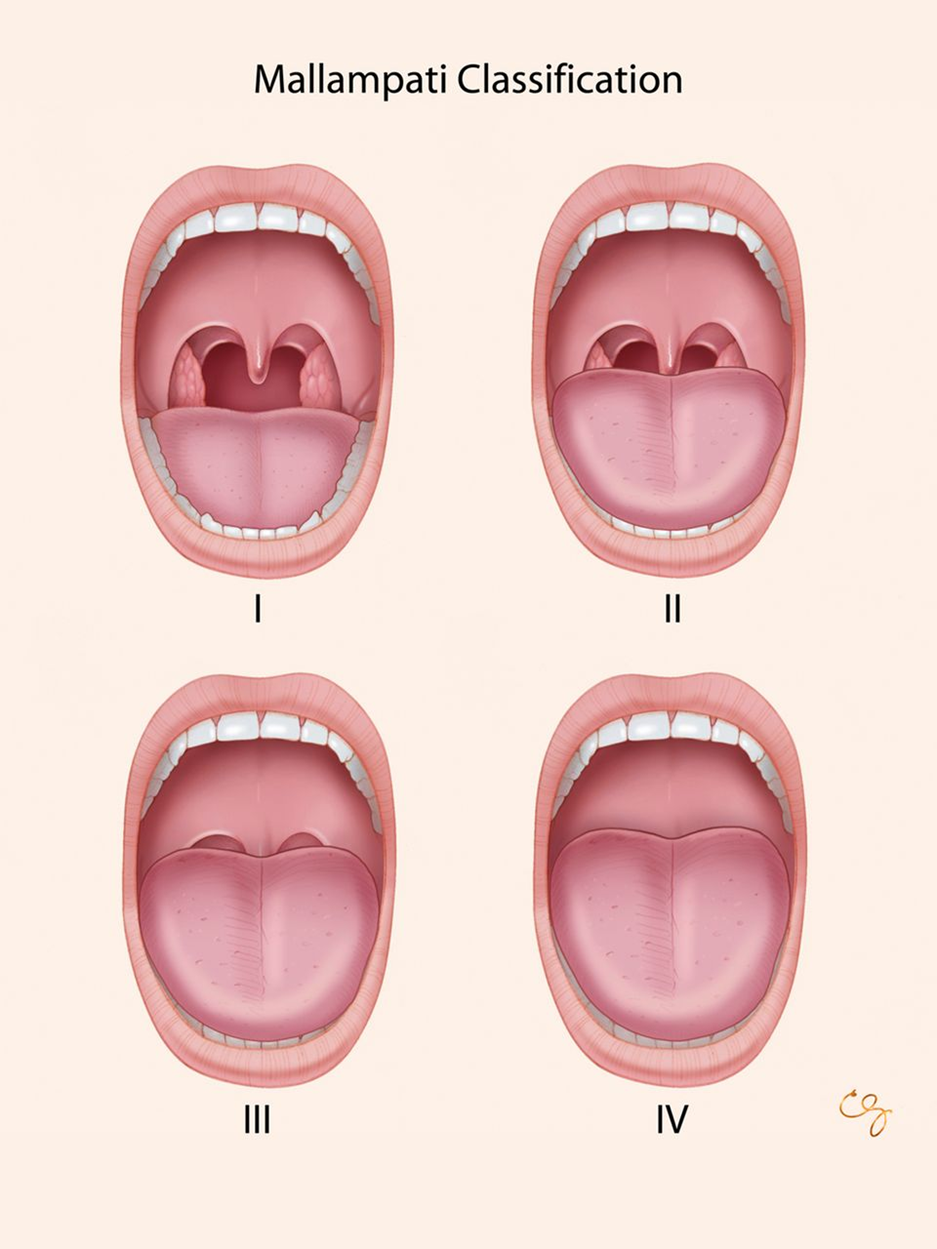


With regard to airway assessment, which term best describes this image?

1. Mallampati 1
2. Mallampati 2
3. Mallampati 3
4. Mallampati 4
5. Mallampati classification is not applicable

2.
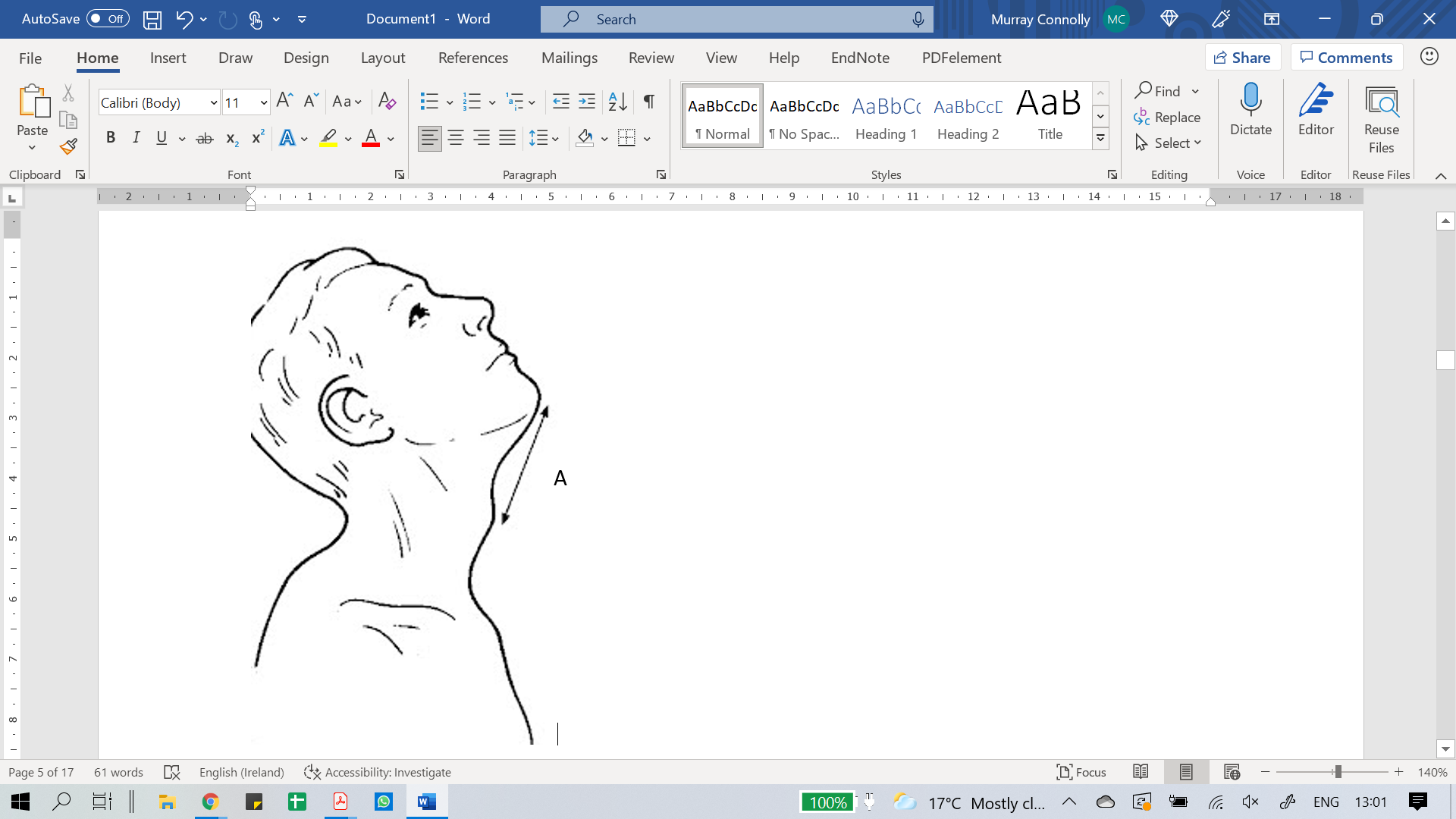


Regarding airway assessment, which sentence best describes the classical cut-off which may predict difficult airway management?

1. Distance A greater than 8 centimetres
2. Distance A less than 8 centimetres
3. Distance A less than 6 centimetres
4. Distance A greater than 6 centimetres
5. Distance A greater than 4 centimetres

3. The next patient on the operating list is a sixty-two year old man scheduled for a laparoscopic cholecystectomy. He has a background history of non-insulin-dependent diabetes mellitus, obstructive sleep apnoea and obesity. On examination he has a BMI of 42, Mallampati Class of 3, a thyromental distance of five centimetres, an inter-incisor distance of 5 centimetres and has reduced neck flexion and extension. Which of these variables is not an indication that his airway may be difficult to manage when anaesthetised?

1. Mallampati class 3
2. Inter-incisor distance of 5 centimetres
3. BMI 42
4. Reduced neck movement
5. Thyromental distance of 5 centimetres

4. Your next patient on the trauma list is a seventy-five year old man who fractured his forearm while out walking. He has a history of hypertension, a myocardial infarction two years ago and he is a smoker. He has previously had an uneventful GA for a laparoscopic appendicectomy when he was seven. What is his ASA Classification?

1. ASA 4E
2. ASA 2
3. ASA 3E
4. ASA 3
5. ASA 2E

5. You are the surgical intern admitting a fifty-eight year old patient for a mastectomy. She reports that the last time she underwent a general anaesthetic for a laparoscopic appendicectomy she was told afterwards that she had “aspirated after being put asleep” which necessitated a prolonged post-operative hospital stay. Which of the following conditions would NOT commonly increase the risk of aspiration of gastric content after induction of anaesthesia?

1. Poorly controlled hypertension
2. Acute cholecystitis
3. Fasting for solids for less than six hours
4. Obesity
5. Pregnancy

6. A 65 year old lady is scheduled for a wire guided WLE of her left breast. She weighs 45 kg, and she smokes 25 cigarettes per day but has no medical comorbidities. She is edentulous and following induction of anaesthesia, bag mask ventilation is difficult. Which of the following would you use first to optimise bag mask ventilation?

- 1. A bougie
  2. A supraglottic airway
  3. A laryngoscope
  4. An oropharyngeal airway
  5. An oxford pillow

7. A 74 year old man is being pre-assessed for a radical prostatectomy. His medical history includes hypertension, hypercholesterolaemia and cardiac failure with reduced ejection fraction. He states that he has significant shortness of breath on walking short distances and is comfortable only at rest. What New York Heart Association Functional Classification does he fit?

1. Class 1
2. Class 2
3. Class 3
4. Class 4
5. The NYHA Classification is not applicable in this situation

8. You are the surgical intern in the Pre-Anaesthetic Assessment Clinic. A patient states that the maximum level of exertion they can complete is climbing one flight of stairs. What number of METs (Metabolic Equivalent of Tasks) does this correlate to?

1. 1
2. 2
3. 4
4. 8
5. 10

9. A 35-year-old man is scheduled for incision and drainage of a submandibular abscess under general anaesthetic. A detailed Airway Assessment reveals a large submandibular swelling, a thyromental distance of seven centimetres, an inter-incisor distance of two centimetres, and reduced neck extension. Which of the following is not predictive of difficult airway management?

1. Thyromental Distance of 7cm
2. Inter-incisor distance of 2 cm
3. Reduced neck Extension
4. Submandibular swelling
5. Mallampati score of 4

10. You are pre-assessing a 70 year old woman who has presented for a wide local excision of a breast mass. Their past medical history includes a 40 pack-year smoking history, hypertension and gastro-oesophageal reflux disease. Which of the following is a relative contraindication to managing her airway with a Laryngeal Mask Airway?

1. Age> 65
2. Smoking History
3. Hypertension
4. Gastro-Oesophageal Reflux Disease
5. Breast Surgery
