# Supplementary material for: Delivering clinical tutorials to medical students using the Microsoft HoloLens 2: A mixed-methods evaluation
Source: BMC Med Educ. 2024 May 4;24:498. doi: 10.1186/s12909-024-05475-2 (PMC11070104; doi:10.1186/s12909-024-05475-2)
Supplement: Supplementary file 4 — Additional file 4. [file 12909_2024_5475_MOESM4_ESM.docx]

Additional File 4

**Data Interpretation Examination**

You are the Intern in the Pre-Admission Assessment Clinic. A 70-year-old man is scheduled for an open left radical nephrectomy for a large renal cell tumour in two weeks’ time. He has a long-standing history of atrial fibrillation, heart failure, chronic kidney disease and epilepsy. He is taking warfarin, ramipril, bisoprolol and levetiracetam.

The patient’s Full Blood Count, Coagulation and Urea and Electrolyte results are as follows:

Hb 12.2 g/dl (normal range 13.5-17.5)

WCC 8.3 (normal range 4-11)

Platelets 160 (normal range 150-400)

INR 2.2 (normal range 0.8-1.2)

Na 140 mmol/l (normal range 135-145)

K 4.5 mmol/l (normal range 3.5-5)

Urea 10.0 mmol/l (normal range 2.5-6.7)

Creatinine 180 µmol/l (normal range 60-100)

**5A. Upon questioning the patient states that he becomes breathless upon minimal exertion, he cannot walk up a flight of stairs without stopping and is comfortable only at rest. Please classify his exercise tolerance using the New York Heat Association (NYHA) Scale. What implications does this have regarding both his intraoperative and postoperative course? (3 marks – 1 each)**

- NYHA 3
- High risk of intraoperative complications (accept any appropriate intraoperative complication such as hypotension, CVA, arrhythmia, MI, also accept need for invasive monitoring such as Art line/ CVC as implication of increased risk)
- High risk of postoperative complications (accept appropriate pulmonary or cardiac complications, VTE, CVA, delirium or other appropriate complication)

**5B. What further investigations would you request in this patient given his cardiac comorbidities? (1 mark – 0.5 each)**

- ECG
- Echo

**5C. Regarding each of the patients’ medications, what instructions would you give him regarding stopping or continuing the medications preoperatively? (2 marks – 0.5 each)**

- Warfarin – Stop 5 days in advance of surgery. (Accept 3 Days also)
- Ramipril – Do not take morning of surgery
- Bisoprolol – Continue to take on day of surgery
- Levetiracetam – Continue to take on day of surgery

**5D. After stopping his Warfarin for the appropriate time, what blood test will he require preoperatively? What is the target level for this blood test prior to anaesthesia and surgery? (1 mark, 0.5 each)**

- INR (also accept coagulation studies)
- INR <1.5
