# Supplementary material for: Delivering clinical tutorials to medical students using the Microsoft HoloLens 2: A mixed-methods evaluation
Source: BMC Med Educ. 2024 May 4;24:498. doi: 10.1186/s12909-024-05475-2 (PMC11070104; doi:10.1186/s12909-024-05475-2)
Supplement: Supplementary file 5 — Additional file 5. [file 12909_2024_5475_MOESM5_ESM.docx]

Additional File 5

**OSCE Examiner, Simulated Patient and Candidate Instructions and Marking Scheme**

| **Examiner Instructions**  **This is a 9-minute station**  The aim of the station is to assess the candidate’s ability to:   - **Perform a focused Pre-Anaesthetic Airway Examination**   The students should be able to:   - **Perform the necessary steps in airway assessment** - **Demonstrate ability to interpret airway exam findings** - Please carefully read the candidate instructions and the answer sheet - Use pencil provided to complete the Mark Sheet - Complete a mark for every question and use the eraser to correct any errors - Greet the student and verify their identity with the corresponding candidate details on the mark sheet. No need to put sticker on the examination sheet - The candidate is given **1 minute to read their instructions** for that station, after which there will be a general announcement telling them when they can approach the simulator. - During the exam ask the following questions: - ***(only ask if exam performed): what is a normal range for neck movement?*** - ***(only ask if exam performed): What is a normal value for mouth opening?*** - ***(only ask if exam performed): What Mallampatti Grade is the patient?*** *Note: If genuinely ambiguous grade, for example 3 or 4 then accept either or the range* - ***(only ask if exam performed): What is a normal distance?*** *Note: some literature mentions 6.5 or 7.5 cm, also accept these values* - **A bell will ring at 4 minutes and 1 minute before the end** - If the candidate finishes the station early, please ask them to sit quietly until the bell goes. However, they may ask further questions if they wish. |
| --- |

**Global Grade Descriptors**

| **Station 2**  **Station Title: Anaesthesia** |
| --- |
| ***Clear Fail:***   - Little idea of how to approach the station - Unprofessional behaviour - Disorganised approach, no evidence of planning – tends to random actions, process and questions - Unable to synthesize findings, or recognise abnormal findings - Struggles/no response to questions about applied knowledge   ***Borderline***   - Able to commence station, but often uncertain, and struggles to proceed to completion - Patchy knowledge - Some organisations of approach, but ‘formulaic’ with no flexibility (e.g. ‘lists’ of questions for patients) - Unsystematic / disordered approach - No evidence of reasoning/discrimination when answering questions in the station (e.g. no knowledge of abnormal values)   ***Clear Pass***   - Systematic overall approach to station/task - Demonstrates sufficient organization to permit completion of task with some evidence of flexibility of approach - Able to summarize (e.g. present history/explain) and manage additional questioning with evidence of reasoning |

| **Candidate Instructions**   - You are the surgical intern manning the anaesthetic pre-assessment clinic. - The next patient due to be seen is scheduled for a radical nephrectomy in three weeks’ time.   **Instructions**   - Please perform a focused **Pre-Anaesthetic Airway Examination** - During the station, the examiner will ask you questions or prompt you to do things - Please explain what you are doing as you do it - **You have 1 minute to read instructions and 9 minutes to complete the station.** - **A bell will ring at 4 minutes and 1 minute before the end** |
| --- |

**Simulated Patient Instructions**

| **Station 2**  **Station Title: Anaesthesia** |
| --- |
| **Offer the following information when asked why you have come to see your doctor today**:  You have been recently diagnosed with a kidney tumour that needs taking out.  **Past Medical/Surgical History (give this information only if asked):**   - No medical issues - Appendix taken out as a child.   **Anaesthetic history**: no recollection of sore throat/ nausea & vomiting after your anaesthetic as a child. No family history of trouble with having an anaesthetic.  **Meds:** None |

**OSCE Marking Scheme**

| **I = Inadequate, A = Average, G = Good, V = Very Good** | **I** | **A** | **G** | **VG** |
| --- | --- | --- | --- | --- |
| **Introduce** self/**Identify** patient (Name and Age); **Explain he/her is** going to ask some questions/perform PE; **ask for permission**; **Hand Hygiene** *(****G****= if also explain and ask permission)* | **0** | **2** | **3** |  |
| **General Inspection** *(****A:*** *Comment appropriately on (lack of) abnormalities from end of bed,* ***I:*** *does not)* | **0** | **1** |  |  |
| **Neck Movement:** Observes patient from the side, requests full flexion and extension; answer *≥ 90 degrees (****VG****= if answer correctly;* ***G:*** *for 2,* ***A:*** *Only requests full flexion and extension)* | **0** | **2** | **4** | **5** |
| **Mouth Opening/ Inter-incisor distance:** Asks patient to open mouth fully, Measures distance between incisors (fingers or ruler); *4cm/ 3 fingers* *(****VG****=if answer correctly;* ***G:*** *all;* ***A*** *only asks to open fully and does not measure)* | **0** | **2** | **4** | **5** |
| **Mallampatti Score:** Asks patient to open mouth fully and protrude tongue; Can use pen torch but not necessary; knows score *(****VG****= answer score;* ***G:*** *Both open mouth and protrude tongue,* ***A:*** *Open Mouth only)* | **0** | **2** | **4** | **5** |
| **thyromental Distance:** Ask patient to extend neck, measure tip of chin to thyroid notch (fingers or ruler); normal distance *≥6cm* (VG= if answer correctly; ***G:*** *Both, A: measures without extension)* | **0** | **2** | **4** | **5** |
| **Ability to Prognath (the upper lip bite test):** Examines patient from the side, asks to protrude mandible or bite top lip with bottom teeth *(****G:*** *Both,* ***A:*** *does not examine from side)* | **0** | **2** | **4** | **5** |
| **Give Summary:** *(****G****: Gives accurate summary of findings and interprets if abnormal,* ***A****: Summarises without interpretation,* ***I****: Neither)* | **0** | **1** | **3** |  |
